# Evaluation of the effect of tartrazine on the offspring rats in an in vivo experimental model

**DOI:** 10.1002/fsn3.4485

**Published:** 2024-09-25

**Authors:** Osman Öztürk, Yusuf Dikici, Öznur Gür, Mert Ocak, Züleyha Doğanyiğit, Aslı Okan, Evrim Suna Arıkan Söylemez, Şükrü Ateş, Sümeyye Uçar, Mustafa Unal, Seher Yılmaz

**Affiliations:** ^1^ Department of Pediatrics, Faculty of Medicine Yozgat Bozok University Yozgat Turkey; ^2^ Department of Mechanical and Aerospace Engineering Case Western Reserve University Cleveland Ohio USA; ^3^ Department of Mechanical Engineering, Faculty of Engineering, Architecture and Design Bartın University Bartın Turkey; ^4^ Department of Mechanical Engineering Karamanoglu Mehmetbey University Karaman Turkey; ^5^ Department of Basic Medical Sciences, Anatomy, Faculty of Dentistry Ankara University Ankara Turkey; ^6^ Department of Histology and Embriology, Faculty of Medicine Yozgat Bozok University Yozgat Turkey; ^7^ Department of Medical Biology, Faculty of Medicine Afyonkarahisar Health Sciences University Afyonkarahisar Turkey; ^8^ Department of Anatomy, Faculty of Medicine Yozgat Bozok University Yozgat Turkey; ^9^ Department of Anatomy, Faculty of Medicine Erciyes University Kayseri Turkey; ^10^ Department of Bioengineering Karamanoglu Mehmetbey University Karaman Turkey; ^11^ Department of Biophysics, Faculty of Medicine Karamanoglu Mehmetbey University Karaman Turkey

**Keywords:** azo dye, bone microstructure, bone quality, Raman spectroscopy, tartrazine

## Abstract

Tartrazine, an azo dye prevalent in pharmaceuticals and food items, was investigated for its impact on fetal development, specifically examining visceral and skeletal abnormalities in rat offspring exposed to daily oral doses throughout pregnancy. Fourteen pregnant rats were randomly assigned to control and tartrazine groups (seven animals each), with tartrazine administered via oral gavage at 7.5 mg/kg throughout gestation. Offspring were categorized by gender for histopathological and genetic analysis of visceral structures. Bone quality and fracture resistance assessments involved micro‐CT, Raman spectroscopy, and biomechanical testing. Results highlighted distinct internal organ tissue differences in the tartrazine group, notably increased hemorrhagic and inflammatory cell infiltration, degeneration, and vacuolization compared to controls. Gender‐specific alterations in mRNA levels of *IL‐6*, *IL‐1β*, *TNF‐α*, and *TRPM2* genes (*p* < .001) were also noted. Moreover, tartrazine‐exposed groups exhibited reduced trabecular thickness, bone volume, and significant alterations in bone matrix composition and quality alongside significant decreases in fracture resistance (*p* < 0.05). This study concludes that intrauterine exposure to tartrazine can result in adverse impacts on organ and bone development in rat offspring.

## Graphical abstract

During pregnancy, tartrazine can cross the placenta and cause various morphological and microstructural changes in the organs and bones of the offspring. It also has effects on the biomechanical structures of bones.

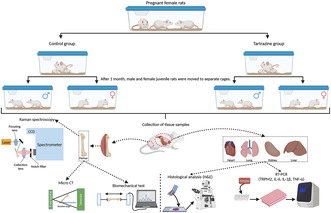

## 1 INTRODUCTION

Tartrazine, a synthetic yellow food dye widely used in industries like food and textiles (Ismail & Rashed, 2022), serves to enhance and preserve flavor, color, and texture in food items (Amchova et al., 2015). Such artificial food colors undergo approval from the Food and Drug Administration (FDA) before integration into the food industry (Dey & Nagababu, 2022), with tartrazine ranking among the most prevalent dyes (El‐Desoky et al., 2022). To regulate concentrations in food, cosmetics, and pharmaceuticals, the acceptable daily intake (ADI) for humans ranges from 0 to 7.5 mg/kg body weight (Pressman et al., 2017).

Tartrazine, a common additive, is found in various daily food products such as chips, fruit juices, cakes, cornflakes, soups, candies, ice creams, and gums. Additionally, it is utilized in shampoos and hair care items (Barciela et al., 2023; Pay et al., 2023). Studies have associated tartrazine with toxic effects stemming from metabolic processes that transform its azo bond (Al‐Seeni et al., 2018; Haugabrooks & Hayes, 2023). Some research also hints at potential carcinogenic effects, particularly concerning intestinal absorption and these effects appear to vary based on an individual's age, gender, and genetic disposition (Hanna et al., 2023; Sambu et al., 2022).

The metabolites of tartrazine instigate the production of reactive oxygen species (ROS), precipitating oxidative stress that exerts biochemical and cellular repercussions on organs such as the liver, stomach, and kidneys (Albasher et al., 2020; Amin et al., 2010; El Golli, 2016; Visweswaran & Krishnamoorthy, 2012). These metabolites, when transformed into aromatic amines, can traverse both the blood–brain and placental barriers. Research indicates potential teratogenic effects from food additives consumed during pregnancy (Hashem et al., 2019). There are also several studies on embryotoxic substances during pregnancy, exploring fetal bone development using diverse staining methods (Tokpinar et al., 2024; Yılmaz et al., 2020). Previously, embryotoxic and teratogenic effects of tartrazine have been investigated in various animal models. For example, previous studies have shown that tartrazine exhibits teratogenic effects on rats and zebrafish embryos (Gupta et al., 2019; Hashem et al., 2019; Sambu et al., 2022). Additionally, it has been reported to have genotoxic potential and induce DNA damage in the liver, kidneys, and leukocytes of rats (Balta et al., 2019; Khayyat et al., 2017; Nasri & Pohjanvirta, 2021). Additionally, Khayyat et al., 2017 found that tartrazine may cause structural and functional aberrations, as well as severe histopathological and cellular alterations in the liver and kidney tissues of rats. These findings suggest potential risks associated with tartrazine exposure during embryonic development and its adverse effects on the physiological functions of various organs. Furthermore, tartrazine has been linked to oxidative stress, elevated hepatocellular enzyme activities, and alterations in kidney biomarkers, indicating potential toxicity in adult rats (El Golli, 2016). Moreover, the impact of tartrazine on learning and memory functions in animals has been studied, suggesting potential neurotoxic effects (Gao et al., 2011). Moreover, Pan et al., 2011 characterized the interaction between tartrazine and serum albumins, revealing conformational and microenvironmental changes that could potentially impact the physiological functions of these proteins.

In the context of bone strength and quality in offspring born to pregnant rats exposed to tartrazine, there is currently no direct evidence linking tartrazine to bone tissue effects. The emerging understanding of its teratogenic and toxic effects on various organs raises concerns about its potential impact on both bone development and several organs. Hence, there exists a discernible necessity for inquiries aimed at unraveling the conceivable impacts of tartrazine on bone fracture resilience and quality, considering the complex interplay between embryonic development and bone synthesis. Consequently, our objective was to scrutinize the influence of tartrazine on bone integrity alongside its effects on other correlated organs in the progeny of pregnant rats subjected to tartrazine exposure.

## 2 MATERIALS AND METHODS

### 2.1 Rats and experimental design

The study utilized 2‐month‐old female Sprague Dawley rats (*N* = 14) weighing 250–300 g obtained from Erciyes University Experimental Animal Center. The appropriateness of this investigation involving animal experimentation adhered to the guidelines outlined in decision 24/008 issued by the Erciyes University Animal Experiments Local Ethics Committee. The rats were fed a standard rat diet. Pregnant rats were accommodated in meticulously arranged chambers equipped with automated heating systems, maintaining a stable temperature of 21°C, and subjected to alternating 12‐hour periods of light and darkness for the duration of the research.

*Control Group (n = 7)*: Physiological saline was administered by oral gavage during pregnancy.

*Tartrazine Group (n = 7)*: Tartrazine at a dose of 7.5 mg/kg/day was administered by oral gavage (El Imam & Abd El Salam, 2018; Ismail & Rashed, 2022) during pregnancy. After the gestation period, the offspring born from the rats were separated by gender and placed in individual. At 1 month of age, they were sacrificed under ketamine xylazine anesthesia. In our study, six male and six female offspring with similar measurements were used for each test.

### 2.2 Sample collection and tissue preparation

**FIGURE 1:**
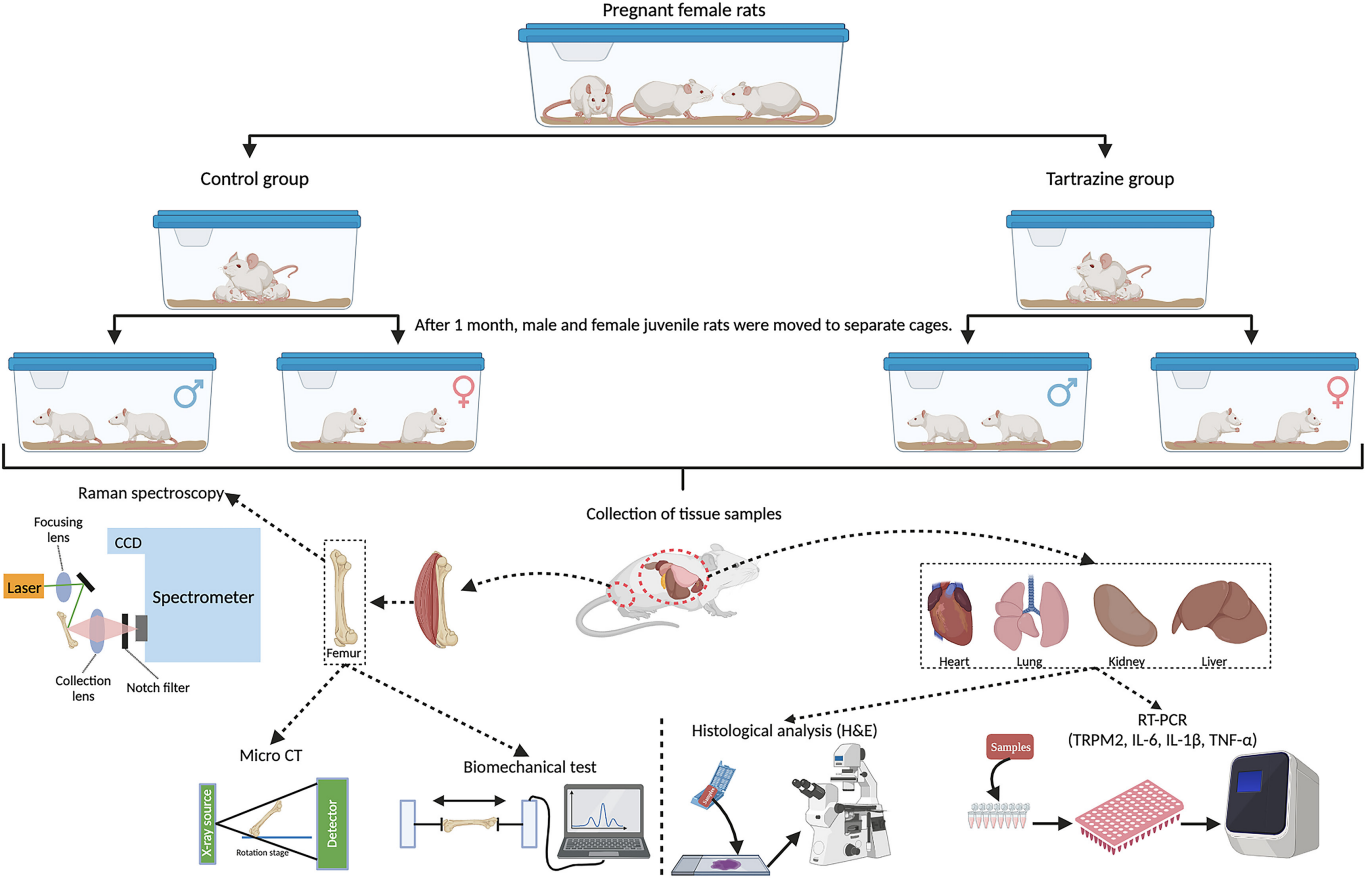
Schematic representation of the experimental design and the parameters examined (Biorender.com).

After sacrifice, heart, lung, kidney, and liver tissues were collected from each animal for histology and Rt‐Pcr analysis. Femur bones were also collected, cleaned with physiological saline (PBS), and stored at −20°C with PBS to prevent dehydration. Samples from each group for each parameter were used for Raman spectroscopy, biomechanical testing, and micro‐CT analysis. The experimental setup and investigated parameters are shown in Figure 1.

### 2.3 Histological analysis

After removing heart, lung, kidney, and liver tissues from both female and male animals, they were placed in a 10% formaldehyde solution and fixed for 8 to 12 hours (Doğanyiğit et al., 2020). After fixation, the tissues were submerged in flowing water for the duration of the night. The routine histological paraffin embedding process was performed on a day. Five‐micrometer‐thick sections were taken from each paraffin block sample. The sections were passed through a xylene and decreasing alcohol series and stained with hematoxylin and eosin. The study evaluated glomerular degeneration, vacuolization in tubulointerstitial injuries, hemorrhage, and infiltration of inflammatory cells in each animal (Inandiklioglu et al., 2021). In liver samples, sinusoidal expansion, bleeding, and apoptotic hepatocytes were evaluated (Doğanyiğit et al., 2020), while in heart samples, bleeding and irregular myocardial fibers were evaluated (Akyuz et al., 2023). In lung samples, hemorrhage and mononuclear cell infiltration were evaluated. Damage rates for each category were scored as follows: 3 severe, 2 medium, and 1 light. No changes were observed in some cases. The tissues were analyzed histopathologically under the Olympus BX53 light microscope.

### 2.4 Genetic analysis

Total RNA was extracted from the hearts, lungs, livers, and kidneys of the tartrazine‐exposed rats using PureZole reagent (Biorad, USA) following the manufacturer's protocol. The quantity and quality of RNA in each sample were assessed employing a Nanodrop ND‐1000 spectrophotometer V3.7. The RNA specimens were preserved at −80°C until required. Subsequently, all RNA samples underwent reverse transcription into cDNA utilizing 1 μg of total RNA (iScript Reverse Transcription Supermix, Biorad, USA). Real‐time PCR was used to analyze the expression of *IL‐6*, *IL‐1β*, *TNF‐α*, and *TRPM2* mRNA. The Step‐One‐Plus Thermocycler (Applied Biosystems) was used for amplification in a total reaction volume of 20 μL, with cDNA, site‐specific primers (Oligomer Biotechnology, Ankara), SsoAdvanced Universal Inhibitor‐Tolerant SYBR Green Supermix (Biorad, USA), and nuclease‐free water. *GAPDH* was used as an internal control. The primer sequences for *TNF‐α*, *IL‐1β*, and *IL‐6* were designed as previously mentioned (Iwashita et al., 2014), while those for *TRPM2* were designed as previously mentioned (Cook et al., 2010).

Rat‐*IL‐6* F: 5′‐TCCTACCCCAACTTCCAATGCTC‐3′

Rat‐*IL‐6* R: 5′‐TTGGATGGTCTTGGTCCTTAGCC‐3′

Rat‐*IL‐1β* F: 5′‐CACCTCTCAAGCAGAGCACAG‐3′

Rat‐*IL‐1β* R: 5′‐GGGTTCCATGGTGAAGTCAAC‐3′

Rat‐*TNF*‐*α* F: 5′‐AAATGGGCTCCCTCTCATCAGTTC‐3′

Rat‐*TNF*‐*α* R: 5′‐TCTGCTTGGTGGTTTGCTACGAC‐3′

*Rat‐TRPM2 F*:5′GAAGGAAAGAGGGGGTGTG‐3′

*Rat‐TRPM2 F*: 5′ CATTGGTGATGGCGTTGTAG‐3′

Rat‐*GAPDH* F: 5′‐ GAGGACCAGGTTGTCTCCTG‐3′

Rat‐*GAPDH* R: 5′‐GGATGGAATTGTGAGGGAGA‐3′

We used the following RT‐PCR protocol: for *IL‐6, IL‐1β, TNF‐α*: 98°C for 3 min of initial denaturation followed by 40 cycles of 98°C for 15 s and 61°C for 30 s, and for *TRPM2*: 98°C for 30 s initial denaturation followed by 40 cycles of 98°C for 5 s and 58°C for 30 s. Melting curve analysis was performed for confirmation of single‐product amplification at the end of the PCR. 65°C–95°C, 0.5°C increments at 5 s/step. Each run has been performed triplicate.

### 2.5 Raman spectroscopy

After the specimens had fully thawed at room temperature, each specimen's long axis was carefully aligned parallel to the primary laser polarization axis. Subsequently, we acquired three Raman spectra per specimen, ranging between 750 and 1800 cm^−1^. These spectra were obtained as an average of ten consecutive spectra per spot, with a 5‐second acquisition duration, utilizing a 20× objective (NA = 0.40) via a 785 nm Raman micro‐spectroscopy (Invia, Renishaw, UK).

Following the established protocols (Unal et al., 2016, 2018, 2021), we calculated various bone composition parameters from the Raman spectroscopy data based on peak intensities (Figure 4a). These parameters include the mineral‐to‐matrix ratio (ν_1_PO_4_/Amide I), type‐B carbonate substitution (CO_3_/ν_1_PO_4_), crystallinity (the reciprocal of the line‐width of the ν1PO4 peak at half‐maximum; 1/FWHM), collagen cross‐links/matrix maturity ratio (~I_1670_/I_1690_), hydroxyproline to proline ratio (Hyp/Pro), and the ~I_1670_/I_1640_ ratio, indicative of collagen conformational changes (Figure 4a) (Unal et al., 2016).

### 2.6 Micro‐CT

Femur samples taken from female and male offspring were fixed with a thin paraffin block. It was aligned in the same direction in the plastic tube for micro‐CT scanning. An isotropic voxel size of 20 μm in a 35 mm field of view was achieved by adjusting the resolution parameters.

Adjusted images were taken on each sample (Bruker Skyscan 1275, Kontich, Belgium). Then, images were obtained through bone tissue with an isotropic voxel size of 10 μm using 40kv, 250 μA, exposure time 49 ms, rotation step 0.2, and 360O rotation in a high resolution 23 mm field of view. Axial, coronal, and sagittal images of each sample were examined using Dataviewer software (Skyscan, Kontich, Belgium). CTAn (Skyscan, Aartselaar, Belgium) software was used for three‐dimensional (3D) volumetric visualization, and area/volume measurement analysis was performed for micro‐CT.

### 2.7 Biomechanical testing

The versatile testing device TA.XT Plus (Stable Micro Systems, UK) was used to conduct the three‐point bending test. The femurs were first placed at two spans with a constant span length of 7.5 mm (Figure 7a) and were loaded to fracture at a rate of 3 mm/min (Creecy et al., 2020). Force measurements were captured using a 500 N load cell, and force and displacement values were automatically recorded using the device's software. The structural‐dependent mechanical properties, such as stiffness, yield, and peak load, were calculated from the force–displacement curve (Figure 7a). The associated beam equations (Jepsen et al., 2015) were utilized in estimating the material properties, including elastic/bending modulus, yield stress, and maximum stress.

### 2.8 Statistical analysis

The numerical values of the histopathological findings were analyzed using a two‐way analysis of variance and Sidak's multiple comparison tests. First, normality of the parameters was tested using a Shapiro–Wilk test. If the normality assumption was met, a one‐way repeated ANOVA test was applied (Doğanyiğit et al., 2024). The genetic analysis was performed using REST 2009 V2.0.13 Software (Pfaffl et al., 2002). Statistical analysis was performed using GraphPad Prism 9 on results obtained from micro‐CT, biomechanical testing, and Raman spectroscopy. Normality and lognormality tests were conducted using the Kolmogorov–Smirnov test. If the normality assumption was met, an unpaired *t*‐test was applied. If the normality assumption was not met, a Mann–Whitney test was applied. The statistical significance level for all comparisons was set at *p* < .05 (Selvan et al., 2024).

## 3 RESULTS

### 3.1 Histology results

**FIGURE 2:**
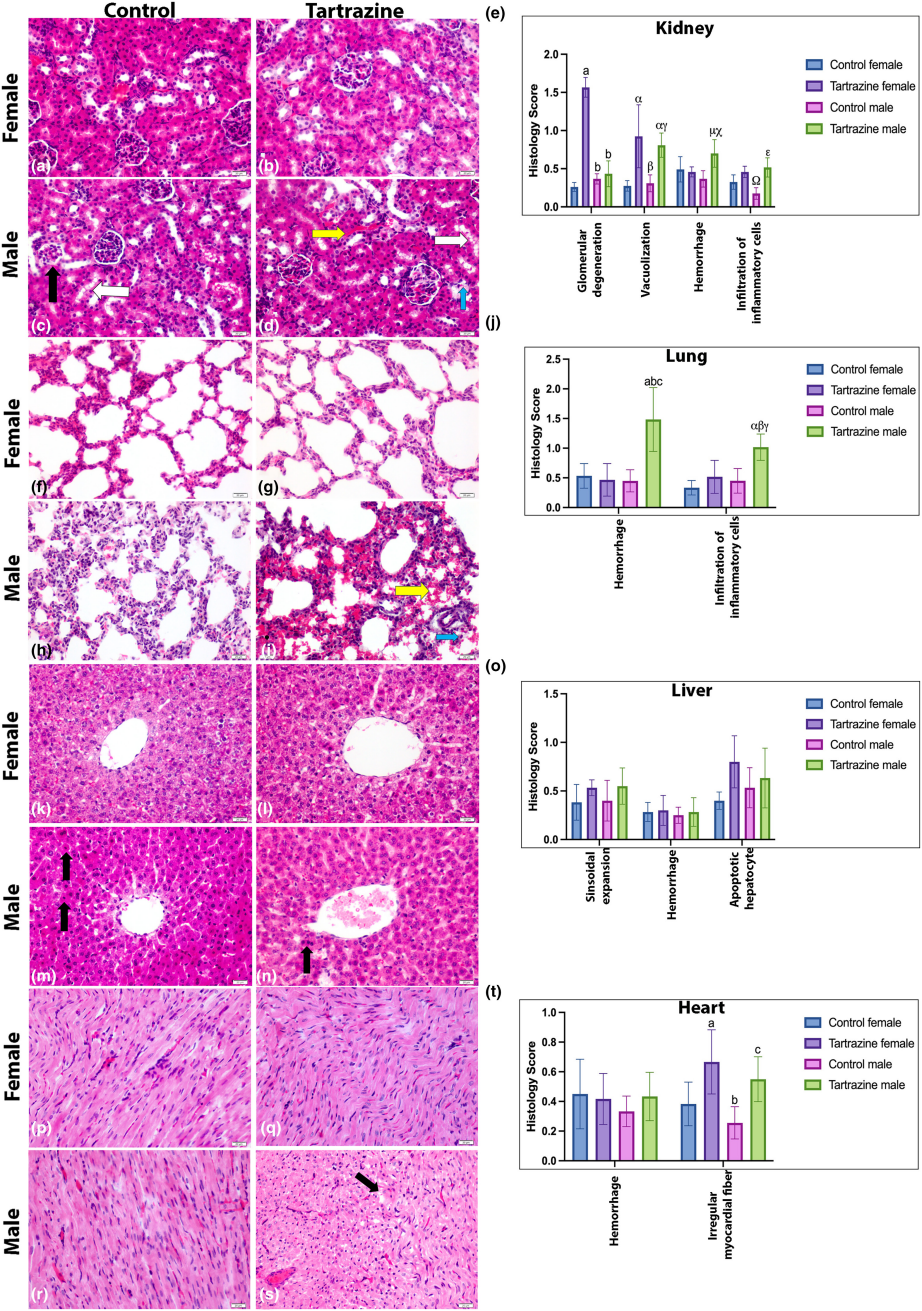
Hematoxylin and Eosin‐stained images of kidney, lung, liver, and heart tissues from both male and female experimental groups. The images include control female group (a, f, k, p), tartrazine‐exposed female group (b, g, l, q), control male group (c, h, m, r), and tartrazine‐exposed male group (d, i, n, s). The magnification is set at 40× with a scale bar of 20 μm. In the kidney images, the yellow arrow indicates hemorrhage, the black arrow points to glomerular degeneration, the white arrow highlights vacuolization, and the blue arrow shows infiltration of inflammatory cells. For lung images, the yellow arrow signifies hemorrhage, while the blue arrow denotes inflammatory cell infiltration. The liver image showcases black arrows indicating apoptotic hepatocytes, and the heart image highlights irregular myocardial fibers. Histology score bar charts for kidney (e), lung (j), liver (o), and heart (t) are provided, expressed as ± SD. The statistical analysis conducted involves two‐way ANOVA and Tukey's multiple comparison tests denoted as follows: (a, α – *p* < .05 different from control female group), (b, β, μ, Ω – *p* < .05 different from tartrazine female group), and (c, γ, χ, ε – *p* < .05 different from control male group).

Our results revealed increased hemorrhaging in the kidneys and lungs of male groups exposed to tartrazine. Additionally, in female renal tissues, there were notable instances of glomerular degeneration and vacuolization. Statistically significant differences were found in vacuolization, hemorrhage, and inflammatory cell infiltration. Notably, tartrazine led to increased hemorrhage and mononuclear cell infiltration in male lung tissues. Moreover, female liver tissues exposed to tartrazine showed a higher count of apoptotic hepatocytes compared to the control group. While examining female heart tissues, there was a notable increase in irregular myocardial fibers compared to the control group (Figure 2).

### 3.2 mRNA analysis of *IL*‐6, *IL*‐1β, *TNF*‐α, and *TRPM2*

**FIGURE 3:**
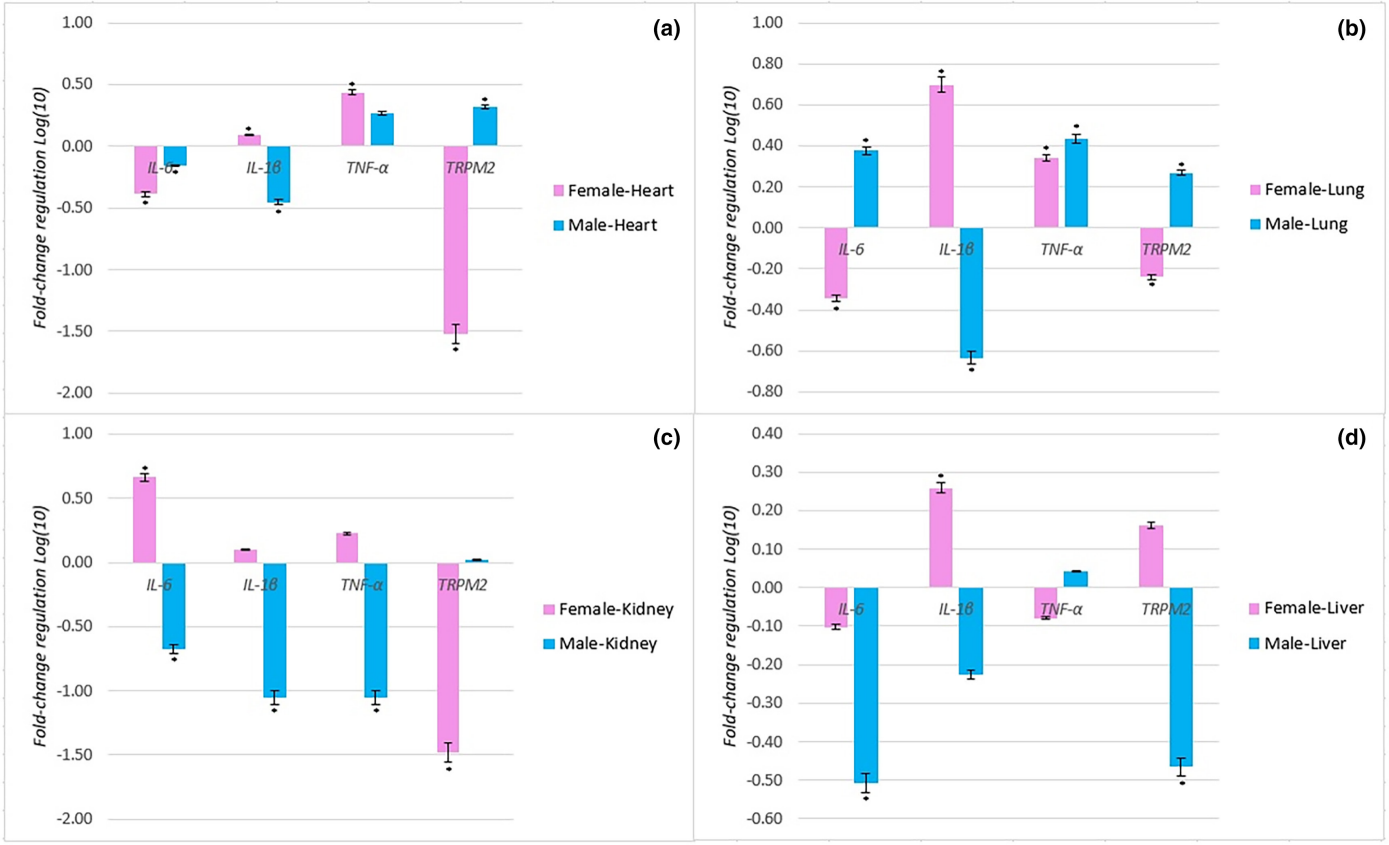
The results of real‐time PCR analysis. Relative mRNA expressions of *IL‐6, IL‐1β, TNF‐α*, and *TRPM2* genes in heart (a), lung (b), kidney (c), and liver (d) tissues exposed to tartrazine were given as fold regulation level, *log(10)*. *Represents the significance of *p* < .001 compared to control. *GAPDH* is a reference gene for normalization.

Alteration of *IL‐6, IL‐1β, TNF‐α*, and *TRPM2* mRNA levels of each tissue of each gender were determined according to the mRNA levels of related control tissues. The mRNA levels of the *IL‐6, IL‐1β, TNF‐α*, and *TRPM2* genes were altered in hearts of females compared to the control (0.41*, 1.23*, 2.73*, 0.03*; fold regulation value, respectively, *p < .001). The mRNA levels of the *IL‐6, IL‐1β, TNF‐α*, and *TRPM2* genes were altered in hearts of males compared to the control (0.7*, 0.4*, 1.8, 2.06*; fold regulation value, respectively, *p < .001). The mRNA levels of the *IL‐6, IL‐1β, TNF‐α* and *TRPM2* genes were altered in lungs of females compared to the control (0.46*, 4.99*, 2.19*, 0.58*; fold regulation value, respectively, *p < .001). The mRNA levels of the *IL‐6, IL‐1β, TNF‐α*, and *TRPM2* genes were altered in lungs of males compared to the control (2.38*, 0.23*, 2.73*, 1.85*; fold regulation value, respectively, *p < .001). The mRNA levels of the *IL‐6, IL‐1β, TNF‐α*, and *TRPM2* genes were altered in kidneys of females compared to the control (4.6*, 1.25, 1.68, 0.03*; fold regulation value, respectively, *p < .001). The mRNA levels of the *IL‐6, IL‐1β, TNF‐α*, and *TRPM2* genes were altered in kidneys of males compared to the control (0.21*, 0.99*, 0.088*, 1.05; fold regulation value, respectively, *p < .001). The mRNA levels of the *IL‐6, IL‐1β, TNF‐α*, and *TRPM2* genes were altered in livers of females compared to the control (0.8, 1.81*, 0.83, 1.45; fold regulation value, respectively, *p < .001). The mRNA levels of the *IL‐6, IL‐1β, TNF‐α*, and *TRPM2* genes were altered in livers of males compared to the control (0.31*, 0.6, 1.1, 0.34*; fold regulation value, respectively, *p < .001) (Figure 3).

### 3.3 Raman spectroscopy

**FIGURE 4:**
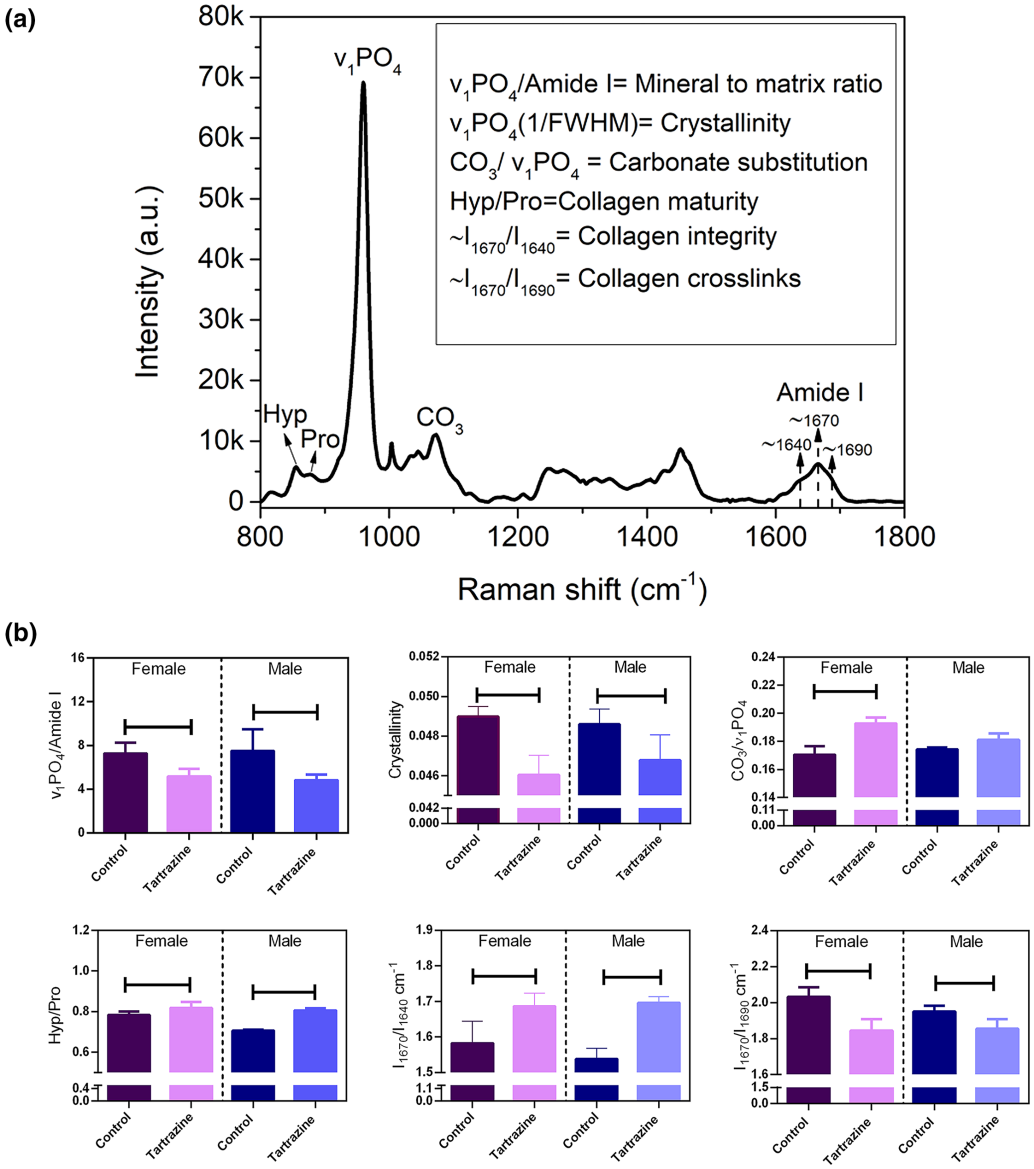
(a) A typical Raman spectrum of bone and calculated Raman spectroscopy‐based bone quality properties. (b) Both mineral and collagen quality properties are significantly affected by tartrazine. The statistical significance level for all comparisons was set at *p* < .05.

Our Raman analysis revealed substantial alterations in bone composition and quality within the tartrazine‐exposed groups compared to the control, regardless of sex. Specifically, the mineral‐to‐matrix ratio (ν_1_PO_4_/Amide I) exhibited a significant decrease in the tartrazine group (Figure 4b), indicating reduced local tissue mineralization. Concurrently, lower crystallinity values in the tartrazine group suggested diminished mineral crystal perfection compared to the control group. Moreover, there was an observed increase in carbonate substitution in the tartrazine groups across both sexes, with statistical significance reached specifically within the female group. This alteration highlights a notable change in bone mineral quality and maturity due to tartrazine exposure. Regarding collagen‐related Raman properties, the Hyp/Pro ratio, indicative of collagen maturity and structural alteration, exhibited a significant increase in the tartrazine group. Similarly, an increase in the ~I_1670_/I_1640_ ratio, reflecting alteration in collagen triple helix structure integrity, was noted in the tartrazine group compared to the control group. Additionally, the mature‐to‐immature enzymatic cross‐links ratio (~I_1670_/I_1690_) significantly decreased in the tartrazine group, suggesting further structural changes in collagen. Our findings underscore the significant impact of tartrazine on bone composition and quality, affecting both mineralization and collagen structure across the studied groups (Figure 4b).

### 3.4 Micro‐CT

**FIGURE 5:**
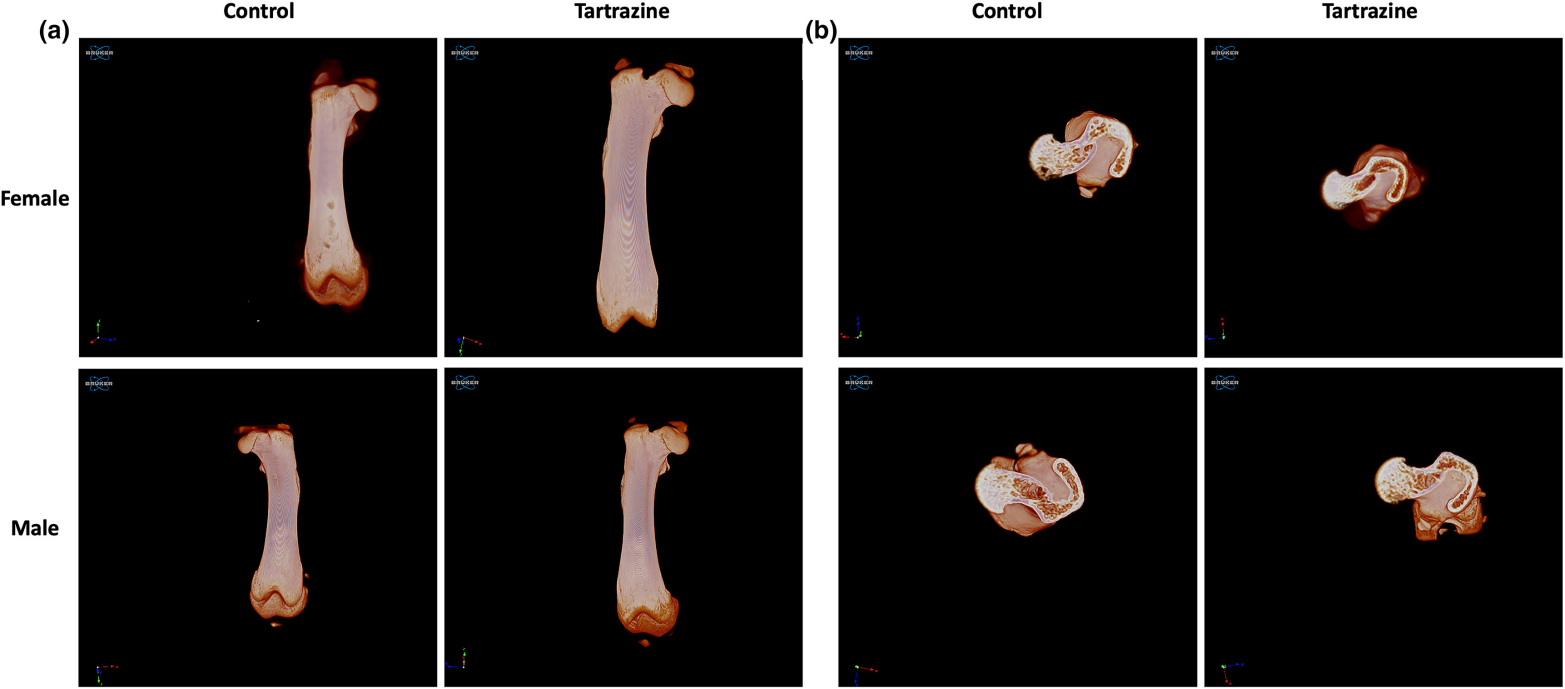
Micro‐CT scan of the femur (a), the micro‐CT images show that the trabeculae and trabecular separating head of the femur and greater trochanter can be seen in transverse section (b).

Figure 5 displays micro‐CT images showcasing the trabecular bone appearance of the total femur and femoral head across all groups. The control female and male groups exhibited similar cancellous bone structures characterized by numerous trabecular connections. In contrast, the tartrazine‐exposed groups displayed numerous trabecular breaks and significantly larger trabecular porosity.

**FIGURE 6:**
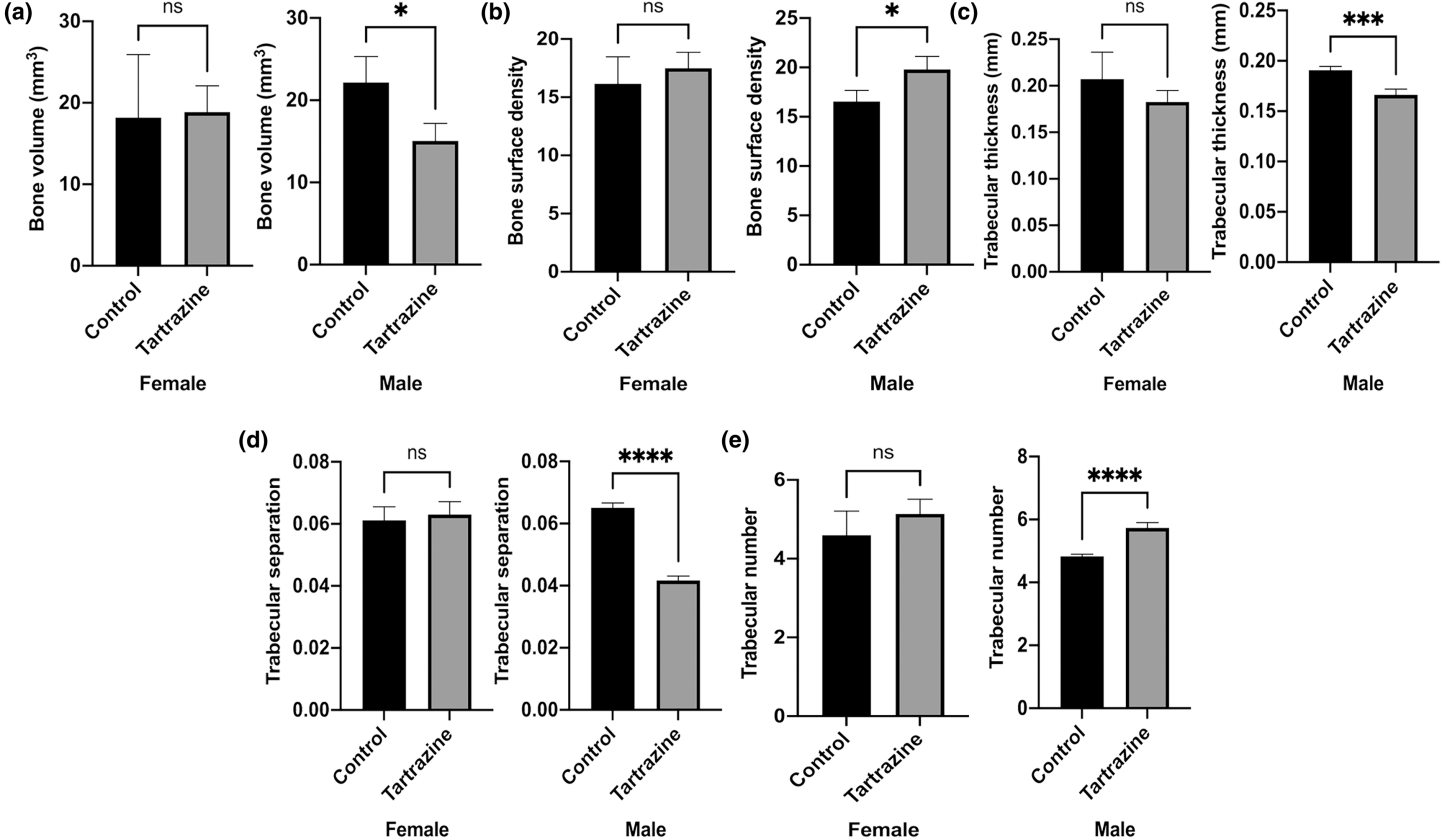
Bone volume (mm^3^) (a), bone surface density (b), trabecular thickness (mm) (c), trabecular separation (d), trabecular number (e). Values are given as the means ± SD. The statistical significance level for all comparisons was set at *p* < .05 (*p < .05, ***p < .001, ****p < .0001, ns; *p* > .05).

Figure 6 highlights key findings: Trabecular thickness in the tartrazine‐exposed groups was notably lower compared to the control group in male (*p* < .001). In females, this value was also lower in the tartrazine‐applied group compared to the control, but the difference was not statistically significant (*p* > .05). Bone volume was reduced in tartrazine‐exposed male offspring in contrast to the control (*p* < .05). Moreover, trabecular separation was observed to be diminished in tartrazine‐exposed male offspring in comparison to the control (*p* < .0001). When bone surface density and trabecular number values were examined, an increase was observed in both males and females as a result of tartrazine exposure compared to the control group. This increase was statistically significant in males (*p* < .05).

### 3.5 Biomechanical testing

**FIGURE 7:**
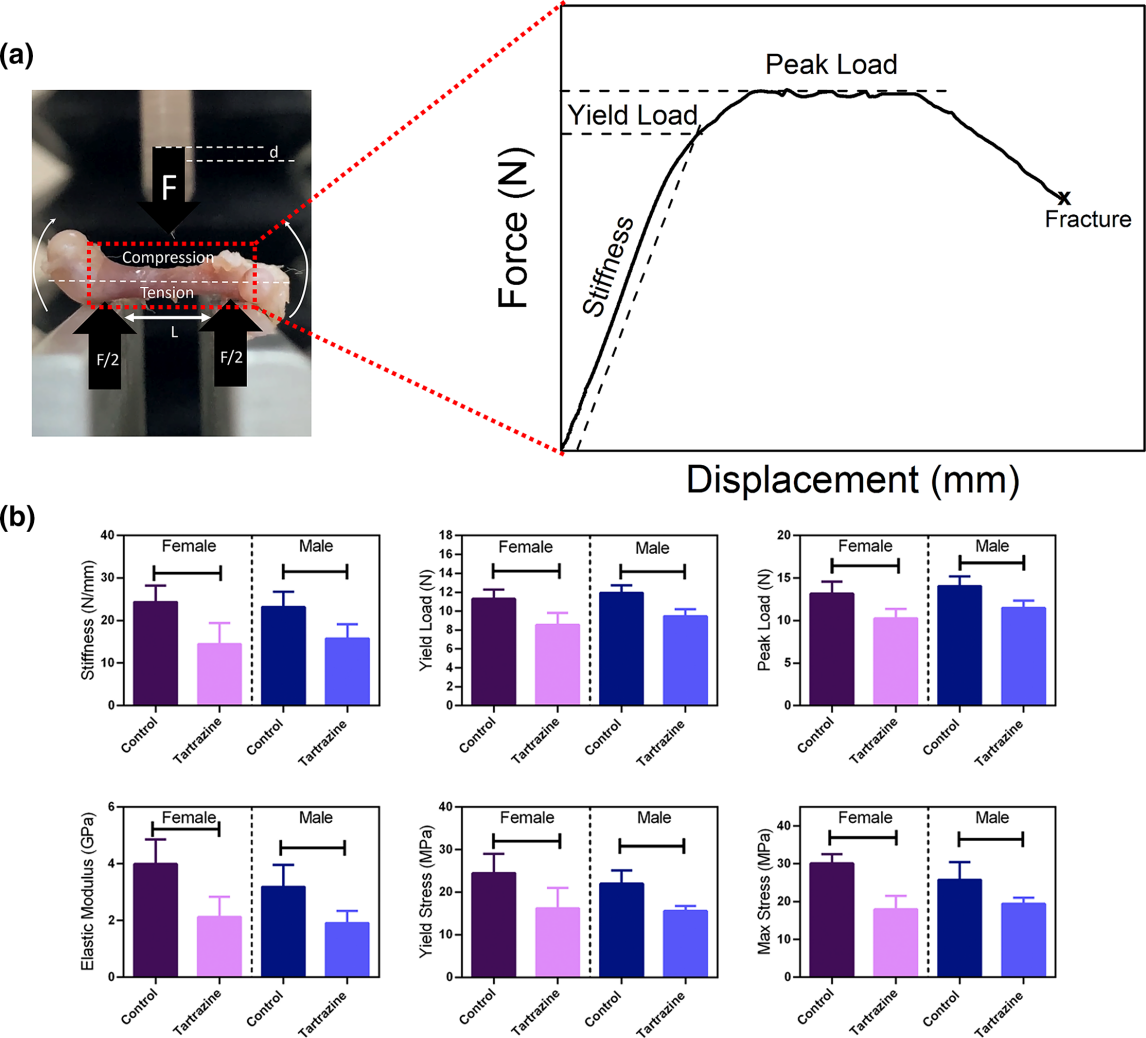
(a) A three‐point bending test conducted on femurs, accompanied by the corresponding typical force–displacement curve. (b) Both structural and material‐level biomechanical properties of femurs exhibited significant reductions in the tartrazine‐exposed groups in comparison to the control groups (*p* < .05).

Our investigation into both structural‐ and material‐level mechanical properties revealed significant reductions in the mechanical fracture resistance of femurs due to tartrazine exposure. Notably, stiffness and its material‐level counterpart, elastic modulus, displayed decreases in the tartrazine‐exposed group compared to the control, irrespective of sex (*p* < .05). Furthermore, both yield load (structural level) and yield stress (material level) exhibited decreases in the tartrazine‐exposed groups in contrast to the control groups (*p* < .05). Both peak load and maximum stress also showed considerable reduction in the tartrazine‐exposed groups when compared to the control group (*p* < .05) (Figure 7b). Collectively, these mechanical findings suggest a significant decline in the mechanical properties of femurs in the tartrazine groups, emphasizing its adverse impact on bone fracture resistance (Figure 7b).

## 4 DISCUSSION

Recent studies have reported that the intake and dosage of tartrazine, widely used in the food industry, similar to Indole‐3‐acetic acid in agriculture, can cause cellular damage in experimental animals, potentially leading to organ damage (Haridevamuthu et al., 2024; Nayak et al., 2024). Moreover, according to the study published by Khayyat et al., tartrazine has been linked to potential hepatorenal and cardiovascular toxicity, causing structural and functional abnormalities, genotoxic effects, and alterations in physiological and biochemical parameters (Haridevamuthu et al., 2024; Khayyat et al., 2017). Studies have also reported severe histopathological and cellular alterations in liver and kidney tissues, DNA damage, and changes in antioxidant enzymes, lipid peroxidation, and lipid profile in animal models (Adele et al., 2020; El‐Hakama et al., 2022; Khayyat et al., 2017). Furthermore, tartrazine has been associated with damage to the gastric mucosa, as reported previously (Elwan & Ibrahim, 2019), and alterations in carotid and aortic stiffness, indicating a potential impact on cardiovascular health (Bruno et al., 2017). Histological examinations of organs revealed increased necrosis and inflammation in the liver and kidney tissues of experimental animals exposed to tartrazine, resulting in organ damage. The specific damages observed in the studies include glomerular necrosis, portal vein level edema, and inflammation (Demircigil et al., 2023; El‐Desoky et al., 2022; Hoc et al., 2006). Heightened levels of *TNF‐α*, *IL‐1β*, and *IL‐6* were reported in brain tissue of rats exposed to tartrazine in recent studies (Demirkol et al., 2012; Essawy et al., 2023). Based on the findings, the application of tartrazine to pregnant rats, even at a low dose of 4.5 mg/kg, resulted in regression in heart, lung, and bone development in fetal organs, as well as impaired fetal growth compared to the control group (Hashem et al., 2019). Herein our results also clearly indicate the adverse effects of tartrazine on the heart, lungs, kidneys, and liver tissues of offspring born to pregnant rats exposed to tartrazine (Figures 2 and 3).

Additionally, studies have shown that external intake of substances such as drugs and food dyes during pregnancy can affect embryonic development during the organogenesis period in mammals, crossing the placental barrier (Atay et al., 2019; Booth et al., 2022). Various studies have explored the safety concerns and technological applications of food colorants, including azo dyes (Jiang et al., 2020; Pay et al., 2023). However, there remains a gap in understanding their specific effects on bone quality and fracture resistance.

To our current understanding, this investigation represents the inaugural examination assessing the impacts of tartrazine on the quality of bone tissue and its resistance to fractures. This current study reports the adverse effects of tartrazine on offspring born from pregnant rats exposed to tartrazine, impacting bone tissue, regardless of gender. More specifically, our results indicate that rats exposed to tartrazine during pregnancy exhibit a decrease in bone composition and quality compared to the control group. Raman spectroscopy‐based assessment of bone matrix provides comprehensive information on bone composition, including both mineral and collagen (Unal et al., 2021). Our results suggest significant adverse effects of tartrazine on skeletal development, affecting not only mineral amount per organic matrix but also mineral quality (Figure 4b). Additionally, collagen quality was also diminished due to exposure to tartrazine during pregnancy (Figure 4b). The adverse effects of tartrazine on bone tissue are not limited to bone composition level. Our findings further demonstrate that bone microstructural properties, which play a crucial role in bone fracture resistance (Hoc et al., 2006), are impacted by tartrazine exposure during pregnancy (Figures 5 and 6). Exposure to tartrazine during gestation can have deleterious effects on the bone composition and microstructure of rat offspring, resulting in reduced bone fracture resistance. This assertion is corroborated by the diminished metrics of diverse bone mechanical characteristics noted in the group exposed to tartrazine, in contrast to the control group (Figure 7b). These findings emphasize the need for further research to evaluate the potential link between tartrazine and reduced bone fracture resistance and quality. Tartrazine may affect bone quality and fracture resistance through various mechanisms, including the generation of ROS and oxidative stress. Several studies have shown that tartrazine induces ROS and oxidative stress (Albasher et al., 2020; Amin et al., 2010; El Golli, 2016; Visweswaran & Krishnamoorthy, 2012). In fact, ROS has been extensively studied in bone biology research due to their impact on bone fracture and health. They play a role in physiological processes such as osteoclastic activity in bone resorption, as opposed to osteoblastic activity, which is active in bone remodeling (Filip et al., 2018; Sugumaran et al., 2023). Studies have demonstrated that ROS are involved in osteoclast differentiation and bone resorption, as well as in osteoblast function and bone formation (Agidigbi & Kim, 2019; Schoppa et al., 2022). ROS have been linked to the regulation of osteoclastogenesis and osteoblastogenesis, indicating their involvement in bone remodeling processes (Domazetovic et al., 2017; Zhu et al., 2022). Additionally, excessive ROS generation has been shown to cause oxidative stress, which can impair bone formation and contribute to bone loss, osteoporosis, and age‐related bone fragility (Chen et al., 2015; Cicek & Cakmak, 2018). Conversely, scavenging of ROS has been demonstrated to inhibit osteoclastogenesis and promote bone regeneration, suggesting a potential therapeutic approach for mitigating the adverse effects of ROS on bone health (Dou et al., 2016). Previous studies have established the significant impact of ROS and oxidative stress on bone health, fracture healing, and bone remodeling processes. Therefore, a potential correlation between the detrimental impacts of tartrazine on diminished bone quality and reduced bone fracture resistance might be linked to ROS and oxidative stress mechanisms.

## 5 CONCLUSION

Taking all into consideration, tartrazine was administered orally to pregnant rats during gestation, which resulted in various morphological, microstructural, and biomechanical changes in the organs and bones of the one‐month‐old offspring. The changes in several organs are consistent with previous studies and were supported by histological and real‐time PCR parameters. Moreover, our study demonstrated the effects of tartrazine on bone tissue of the one‐month‐old offspring exposed to tartrazine through placental passage. This study furnishes substantiation for the clinical prevention and treatment of developmental delays or diseases that may occur due to the placental passage of tartrazine and its effects on offspring. Yet, additional research is still necessary to thoroughly examine the potential mechanism link between tartrazine and reduced bone quality and fracture resistance. Therefore, as a future perspective, it is crucial to comprehensively evaluate the effects of tartrazine exposure both during pregnancy and in newborns post‐exposure using various methods. Additionally, investigating the long‐term effects of low‐ and moderate‐dose, chronic tartrazine intake on bone health and overall development could provide valuable insights.

## AUTHOR CONTRIBUTIONS

**Osman Öztürk:** Validation (equal); writing – original draft (equal). **Yusuf Dikici:** Data curation (equal). **Öznur Gür:** Data curation (equal). **Mert Ocak:** Data curation (equal); formal analysis (equal). **Züleyha Doğanyiğit:** Data curation (equal); formal analysis (equal). **Aslı Okan:** Data curation (equal); formal analysis (equal). **Evrim Suna Arıkan Söylemez:** Formal analysis (equal); methodology (equal). **Şükrü Ateş:** Conceptualization (equal); methodology (equal). **Sümeyye Uçar:** Conceptualization (equal); methodology (equal). **Mustafa Unal:** Data curation (equal); formal analysis (equal). **Seher Yılmaz:** Conceptualization (equal); Formal analysis (equal); Methodology (equal); writing – review and editing (equal).

## CONFLICT OF INTEREST STATEMENT

The authors declare that they have no conflict of interest.

## ETHICAL APPROVAL

It was carried out in accordance with the decision numbered 24/008 taken from the Erciyes University Animal Experiments Local Ethics Committee.

## Data Availability

The data that support the findings of this study are available on request from the corresponding author. The data are not publicly available due to privacy or ethical restrictions.
